# Encouraging recruitment into psychiatry: practical initiatives

**DOI:** 10.1192/bjb.2020.53

**Published:** 2021-02

**Authors:** Neel Halder, Zoé Mulliez

**Affiliations:** 1 St Mary's Hospital, Warrington, Elysium Healthcare, UK; 2 Royal College of Psychiatrists, London, UK

**Keywords:** Education and training, psychological testing, ethics, psychotic disorders, risk assessment

## Abstract

This article highlights key findings from a recent Royal College of Psychiatrists project showing that many UK medical schools are embracing the dual challenge of raising students’ interest in psychiatry and ensuring that all doctors can support patients with mental illness. It focuses on two novel approaches to boosting recruitment into psychiatry: I'm a Medic Get Me Out of Here, an online outreach activity enabling schoolchildren to ask questions of health professionals in real time; and a living library, which creates a safe space for dialogue where topics are discussed openly between human books (professionals) and readers (undergraduate students) to challenge stereotypes. It is recommended that sharing these and other examples of good practice will help all medical schools encourage recruitment in psychiatry more widely.

## Showing initiative – encouraging students to choose psychiatry

Over the past two years, the Royal College of Psychiatrists (RCPsych) has gathered and analysed information from medical schools and students to produce practical guidance on how to enhance the undergraduate experience of psychiatry. After hearing many positive examples of activities and initiatives, as well as finding out what students found less appealing, they identified four key action areas for medical schools: excellence in teaching; high-quality psychiatry placements; leadership from psychiatrists; and psychiatry-based enrichment activities.

### Excellence in teaching

To start with, the focus should be on students’ experiences of psychiatry teaching in the classroom or lecture setting. Many students fed back that they would like psychiatry content to be more prominent earlier and throughout the course. Not only would this reflect how psychiatry is an integral part of medicine (rather than just an add-on), but it would allow students to explore at an earlier stage their potential interest in helping patients recover from episodes of mental illness.

A widely held view was that learning in ‘real-life’ contexts should be further promoted, through simulation teaching, learning videos, case-based discussions, testimonies from people with lived experience as patients or carers, and lectures involving a panel of psychiatrists working in a range of subspecialties. The ‘living library’ initiative outlined later in this article is an example of this.

Students also highlighted the importance of accentuating how psychiatry is unique in its integration of science with a whole-person approach, and the role it has in driving the most exciting medical advances to transform treatments and foster overall health and well-being globally.

### High-quality placements

Providing high-quality psychiatry placements is an intuitively sensible way to increase the number of students interested in it as a career. Of the 792 students who participated in our survey, 52.2% said they were more inclined to choose psychiatry after their placement.

**Fig. 1:**
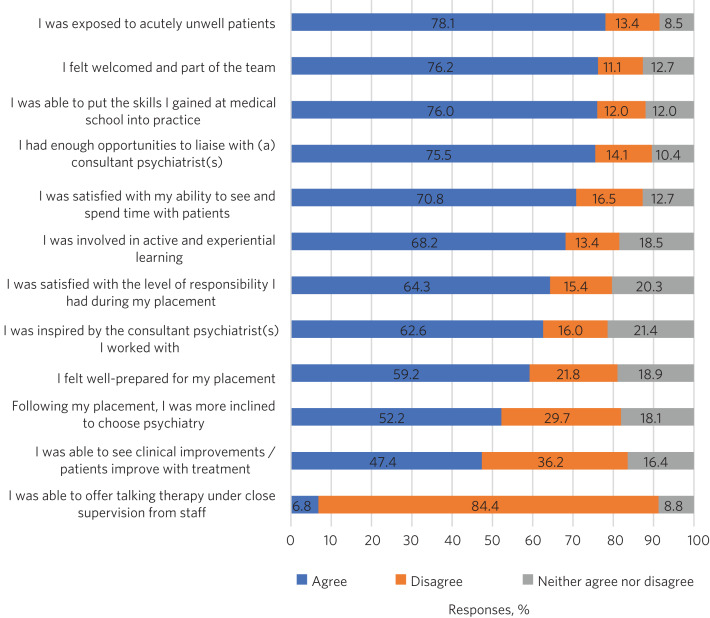
Students’ experiences of placements in psychiatry. A total of 792 students throughout the UK completed the survey. Note: it is rare to find undergraduate settings providing opportunities for undergraduates to offer talking therapy to patients under supervision, as students may not have received enough training to be able to deliver this. Source: unpublished survey run by the Royal College of Psychiatrists between 15 October 2018 and 7 January 2019.

But what does a good placement look like? Fig. 1 illustrates a few components of medical students’ experiences of psychiatry placements.

While there is no singular ‘best’ model for placements, students did report aspects that were more positive.

They particularly enjoyed the variety of experiences provided by psychiatric placements, in contrast to other specialties. Appeal also lies in the ability to talk to patients, more so than in other placements, and learn their history in greater detail. Some medical schools have set up longitudinal placements to provide the opportunity to follow patients during several weeks and observe the longer-term benefits of psychiatric interventions.

Introducing medical students to patients and clinicians in extreme environments such as prisons or secure hospitals arguably has a larger impact on improving attitudes to psychiatry. Archer et al^1^ showed that a single-day visit to Broadmoor Hospital, a high-security psychiatric hospital in Berkshire, was effective in changing the attitudes of medical students towards forensic psychiatry, with 75% of participants stating that their attitude towards psychiatry had improved.

There is a common stereotype of psychiatrists working alone with patients. To combat this, students need to meet all members of the multidisciplinary mental health team. This will ensure that they understand how extensively professionals from a range of disciplines work together to treat patients’ needs holistically.

Students need to understand how psychiatric care fits into the healthcare system and see for themselves the high level of need. One way to do this would be for psychiatric placements to be better combined or coordinated with placements in other specialties and *vice versa*. The students would be able to (a) witness the interactions between general practitioners (GPs), doctors from other specialties and psychiatrists, (b) understand the benefits of integrated psychiatric care and (c) gain further skills in psychiatry, which will be helpful whatever specialty they end up choosing.

How can medical schools help students make the most of their placements? Some students said that they would like to feel better prepared before starting their placements in psychiatry. Budd et al^2^ had previously suggested that this is important, particularly addressing their potential fears and negative perceptions of the specialty. The RCPsych's Choose Psychiatry guidance for medical schools includes suggestions of what inductions might cover based on feedback from students, including: basic guidance on the type of symptoms to expect from patients with different illnesses and how they should respond; a briefing on procedural issues involved in working in psychiatric settings, including health and safety; and information on how a placement in psychiatry might affect their own emotions and mental health (www.rcpsych.ac.uk/become-a-psychiatrist/choose-psychiatry).

Medical students also said that they need reassurance about getting adequate mental health and well-being support. Some medical schools have set up Balint groups to provide a safe space for students to discuss their emotional reactions to their patients’ experiences. These should be supported and further expanded where possible.

### Leadership from psychiatrists

Everyone can remember someone who inspired their career choices. Medical students highlighted the importance of talking to inspiring consultant psychiatrists and trainees during their time at medical school. Some suggested that medical schools could create a ‘bank’ or database of psychiatry mentors.

To raise the profile of psychiatrists in undergraduate education, the RCPsych encourages medical schools to reflect on whether their senior leadership teams are composed of a multispecialty and diverse group of individuals, including psychiatrists, so that students can see them in leadership roles.

Psychiatrists’ progressive and thoughtful leadership could play an integral role in driving the strategic direction of medical schools. The RCPsych advises medical schools to ensure that psychiatric education is designed and led by psychiatrists with both clinical and educational expertise, and that students are presented with up-to-date research in psychiatry throughout the medical curriculum.

### Enrichment activities

How can you help immerse students in psychiatry? Many medical schools have implemented enrichment activities to enhance students’ exposure to and experience of psychiatry, which are highly valued by the medical students we talked to.

Psychiatry societies (‘PsychSocs’) are student-led university societies set up to raise the profile of mental healthcare among medical students and to promote careers in psychiatry. Several next steps are recommended both locally and nationally to take advantage of PsychSocs to improve recruitment into psychiatry – including fostering enthusiastic mentoring by local psychiatrists via ‘buddy schemes’ and continuing to share ideas and learning across the country, as explained by Pandian et al^3^ earlier in 2020.

The Psychiatry Early Experience Programme (PEEP) provides medical students with the opportunity to shadow core trainees in psychiatry. This initiative was developed by South London and Maudsley NHS Foundation Trust and King's College London Medical School. Given the success of the scheme, students asked for improved availability and access to such schemes.

Special study modules (SSMs) or student-selected components (SSCs) in a psychiatry-related subject are short courses and/or projects in subjects that students can select according to their personal interests. They offer opportunities to learn in innovative ways and have been recommended by the General Medical Council.

Career enrichment courses (often referred as summer, autumn or winter schools) offer an intensive programme of lectures, seminars, debates and networking opportunities to students who are considering a career in psychiatry.

Psychotherapy schemes give medical students the unique opportunity to deliver psychotherapy to one patient for an extended period. Yakeley et al^4^ highlighted that projects that involve medical students offering psychodynamic therapy (under the close supervision of staff) have contributed to an increase in the number of students choosing psychiatry as a career.

Extra-curricular initiatives to give students further opportunities to spend time with people with mental illness are also being developed across the country. The Time for Dementia initiative is an example of collaborative work that has had a demonstrable impact on students’ understanding of people with dementia.

Other schemes offered by the RCPsych that can be promoted to medical students include the Psych Star scheme, Student Associate membership, and Divisional and Faculty prizes.

## Case studies: two novel approaches to increase students’ interest in psychiatry

The RCPsych project highlighted that students who were in the early stages of their medical course were more likely than peers later in the course to feel uninformed about psychiatry. Some students in the later years of study also felt that there was a lack of information on mental health and psychiatry and wanted to be better informed.

A key recommendation made by students was to raise awareness of psychiatry at an earlier stage in their medical course and at secondary-school level. The following case studies provide examples of how this recommendation could be implemented.

### A ‘living library’

Greater Manchester Mental Health NHS Foundation Trust decided to use an innovative approach based on the ‘living library’ concept, where experienced clinicians working in their respective fields (‘the books’) were available ‘on loan’ to students (‘the readers’). This was implemented to improve the student experience, enrich students’ learning, provide them with an insight into other professions, encourage the sharing of institutional knowledge and develop interprofessional learning.

The concept is designed to build a positive framework for conversations that can challenge stereotypes and prejudices through dialogue. It is based on the idea of interprofessional learning (defined as ‘when professionals learn with, from and about each other’) that has been identified as an innovative strategy that can help bolster the medical workforce.^5^ Literature reviews suggest that learning with, from and about other healthcare students has the potential to improve communication between professionals and, ultimately, care for patients.^6^

**Fig. 2:**
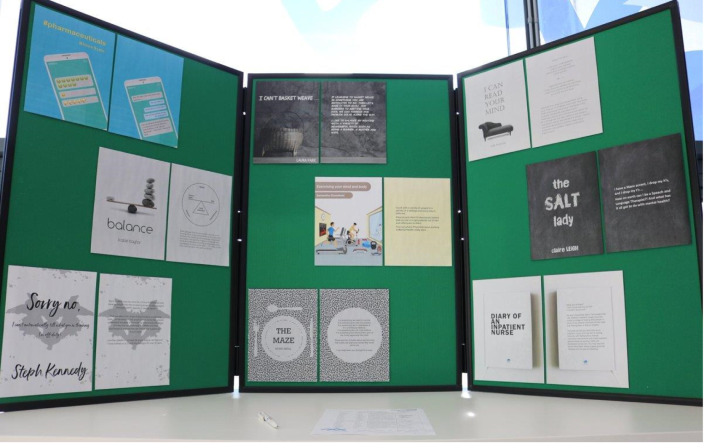
Living library book covers, designed by Lorna Dawson, Greater Manchester Mental Health NHS Foundation Trust.

Figure 2 depicts the book covers that were designed and displayed for students to help decide which ‘book’ they wanted to borrow.

In total, 25 living-library cards were made available to students on placement within the trust.

After signing up to the library and reviewing potential book choices, all readers were given a library card and were invited to reserve one of the books at a slot during the morning. There were six slots available throughout the day and readers would have around 20 min with each of their books. In theory, each reader could have conversations with six of the nine books.

**Fig. 3:**
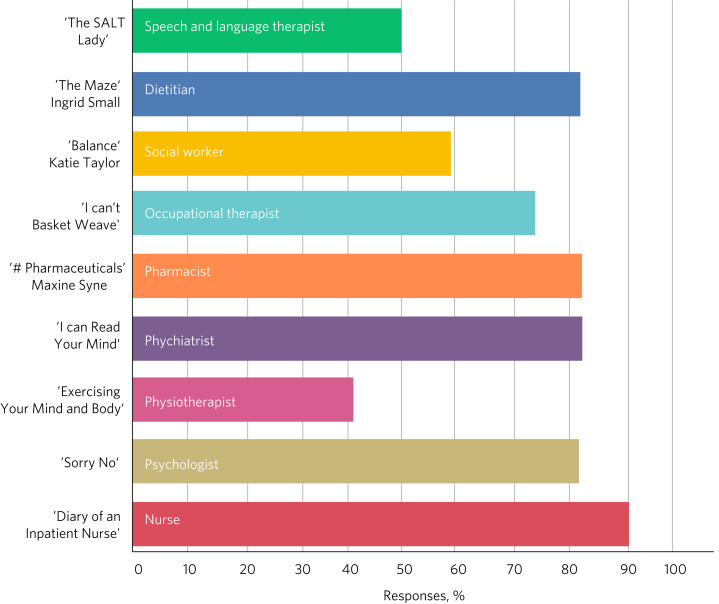
Responses of students (*n* = 13) to the question: ‘Which living book(s) did you read?’.

**Fig. 4:**
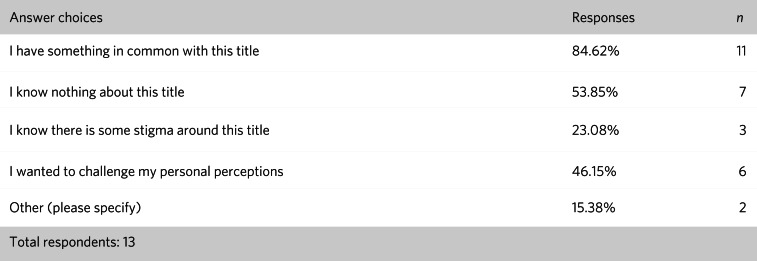
Responses of students (*n* = 13) to the question: ‘What attracted you to the living book(s) you selected?’.

**Fig. 5:**
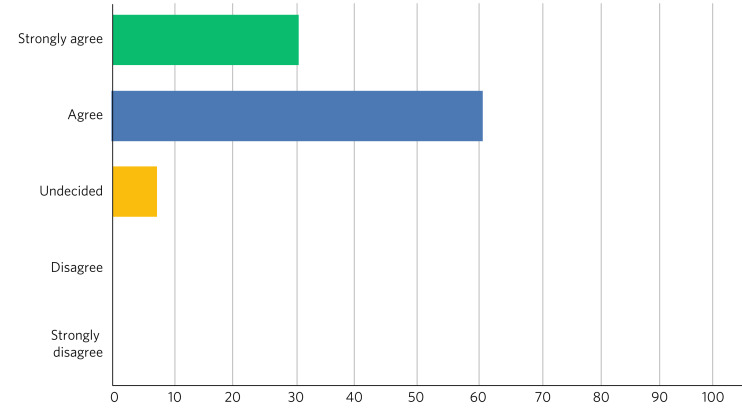
Responses of students (*n* = 13) to the question: ‘Do you feel that your experience at the living library changed your views about another profession?’.

**Fig. 6:**
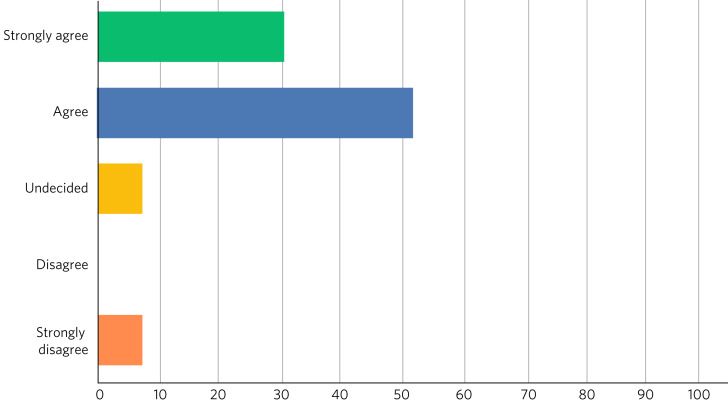
Responses of students (*n* = 13) to the question ‘Has the living library changed your views about the benefits of interprofessional practice?’.

In total, 22 readers booked places on the event, 13 of whom attended. Figure 3 shows which disciplines were the most popular and Fig. 4 describes what attracted the readers to them. Figures 5 and 6 show the impact of the initiative on readers’ views about both professions and the benefits of interprofessional practice.

After the event, 9 (69%) of the 13 respondents rated their overall experience of the living library as ‘Excellent’ and 4 (31%) as ‘Good’; 100% of respondents would recommend living books to others; 11 readers (85%) strongly agreed that the living library was a good way to challenge prejudices and encourage interprofessional learning, with the other 2 (15%) also agreeing with this.

### I'm a Medic Get Me Out of Here

I'm a Medic, Get Me Out of Here (shortened to I'm a Medic) is an online, student-led outreach programme, funded by Health Education England and designed to provide equality of opportunity for all school students to engage with the National Health Service (NHS) workforce. The aim is to help inform schoolchildren about a particular career and let them explore whether that career could be for them. The idea is based on research showing that young people start to develop their career aspirations early in secondary school, if not earlier.^7,8^ Findings from a survey with over 20 000 children showed that parents (and parents’ friends), the TV and media were most likely to influence children's career aspirations. Less than 1% of children had heard about the jobs through people coming to their school.^7^

I'm a Medic was trialled in psychiatry for the first time in 2019. With supervision from teachers, schoolchildren had secure access via a website to healthcare professionals, who answered questions in real time during a 30-min lesson. An online moderator was available for each chat. Pupils could also post a question to be answered at a later stage.

Three healthcare workers took part as individuals: a consultant psychiatrist (N.H.), a mental health nurse and an NHS mental health trust's head of human resources, responsible for managing and advising a wider team. Four healthcare teams based in various locations across England took part as a group: an arts therapy team, an early intervention team, a home treatment team and a psychiatry ward team.

**Fig. 7:**
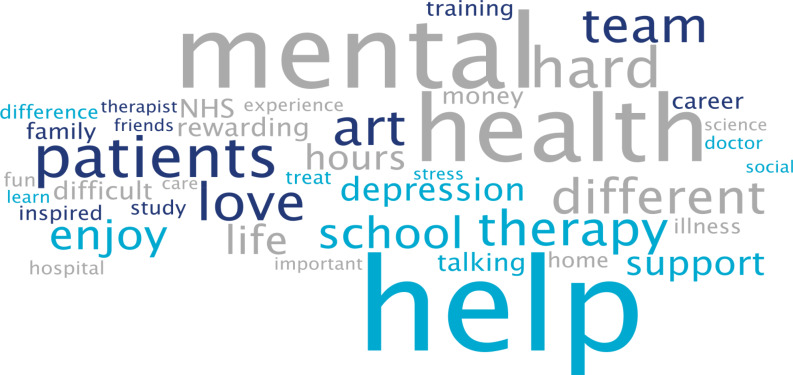
Frequent words used by schoolchildren in live online chats in the I'm a Medic, Get Me out of Here programme. The size of the word represents its popularity.

Students were mainly in year 8 (generally 11.5–13 years old) from schools across England. A total of 47 classes from 20 schools participated in 40 live chats. Over 1000 students logged in, with approximately 85% participating in live chats, asking questions or leaving comments. Students could vote for who they felt they most engaged with and who answered their questions to their satisfaction. Figure 7 depicts the words most often used by students in these conversations.

Students often asked what led healthcare workers to choose their jobs. They would, for instance, ask questions such as ‘What encouraged you to take this job?’ or ‘What inspired you to start what you've done?’.

They also asked healthcare workers about their qualifications and the qualifications they would need to attain certain roles in the sector. These were mostly focused on GCSE qualifications, as opposed to A-Levels.

When discussing mental illnesses, students focused heavily on more commonly known illnesses such as depression and anxiety, including how to discuss or treat them.^9^

During focus groups, students fed back that they particularly enjoyed the ability to interact directly with healthcare workers, in real time and in a ‘conversational’ way. Being able to vote also contributed to the engaging nature of the experience.

Additionally, interacting online provided some distinct advantages that face-to-face interactions might not provide. The first one is the ability for children in remote areas to interact with professionals who may not have visited the schools in person. Second, students often appreciated the opportunity to ask questions anonymously without being judged by their peers:

‘It was better because you're not actually speaking to them. It's, like, all the questions that you ask online you might feel embarrassed to ask them to their face. Then they just find out, because it's easier to type it than to actually say it.’

‘I said some stuff that I would not have said in real life, online. So, it's just easier to, like, speak anonymously.’

This benefit was also highlighted by their teachers:

‘Some of the quieter girls and boys definitely asked a few questions that flagged them up on my radar.’

Data also suggest that the personal and direct nature of the experience helped achieved the desired impact:

‘I think [I connected most with] Neel because of the job that I wanted to be, and he, kind of, helped me, because I'm bad at science, he helped me how to get through it and what qualifications I need. So, that, kind of, helped.’^9^

Fifty responses were collected through a post-survey questionnaire. 82% of the children (*n* = 41) agreed/strongly agreed that they had learnt more about ‘what it's like to work in healthcare’ and felt that they knew more about what they would need to study to get their ideal job; 66% (*n* = 33) agreed that they might get a job working in healthcare, and 60% (*n* = 30) said that they would enjoy working in healthcare. This is an increase from the pre-survey, with responses to those questions being 43% and 45%, respectively.^9^

## Conclusions and future plans

The findings and recommendations detailed in the RCPsych Choose Psychiatry guidance for medical schools and the two case studies included in this article would help lay the foundations for developing a strong medical workforce, comprising both psychiatrists and doctors working in all specialties able to give people with mental illness the best possible care.

The RCPsych project highlighted that students’ consideration of both the importance of mental healthcare in medicine and psychiatry as a career were largely determined by: the integration of psychiatry courses into the curriculum as widely and as early as possible, the high quality of placements in psychiatry, the students’ ability to be in contact with inspiring psychiatry leaders and the availability of enrichment activities to enhance students’ exposure to, and experience of, psychiatry.

Opening up the living library to medical students in the early years of studying could also help increase the number of students choosing to enrol in psychiatry enrichment activities – such as psychiatry modules or psychiatry societies – which in turn will hopefully drive up numbers choosing psychiatry as a career.

We know that schools may have limited resources for careers advice. I'm a Medic is a time-efficient and gratifying initiative that can reach many students without them needing to be taken out of school or disrupting the timetable.

The next step will be to produce a practical booklet to support PsychSocs with their activities. It will include the ideas given above and many others that may not have been considered or shared otherwise, following a consistent framework. The booklet would be particularly helpful to PsychSocs around the UK, but could also be used by foundation doctors and other trainees.

N.H. will contact all UK PsychSocs for contributions but welcomes any authors (from undergraduates to consultants) who wish to contribute. Feedback for this project is also welcome. Please contact the corresponding author.

Meanwhile, the RCPsych is creating an online hub showcasing case studies of psychiatry extra-curricular activities at medical schools across the UK. The case studies will be represented visually on a map of the UK, and users will be able to click to reveal more information about an initiative which will explain how the activity works and may highlight its impact on students’ interest in psychiatry and/or mental healthcare more generally.

Medical schools are also encouraged to use the Gatsby Wellcome Neuroscience Project run by RCPsych to integrate the latest research on neuroscience into their curriculum.

Examples of good practice of how students are being inspired to learn about better mental healthcare have been compiled into a practical guidance published on the RCPsych website as part of the Choose Psychiatry campaign.^10^

## Data Availability

The data that support the findings of this study are available from the corresponding author, N.H., upon reasonable request.

## About the authors

**Neel Halder**, MBCHB, FRCPsych, MSc, CTDip, MBA, is a lead consultant psychiatrist at St Mary's Hospital, Warrington, which is part of Elysium Healthcare. He is also the recruitment lead for the Royal College of Psychiatrists, North West division and an honorary senior lecturer at the University of Manchester, UK. **Zoé Mulliez** is Policy and Campaigns Manager at the Royal College of Psychiatrists. She undertakes policy analysis to inform and fulfil the College's strategic objectives, and manages influencing and public-facing campaigns to secure the best outcomes for people with mental illness. Before joining the College, she worked in policy and research teams in various organisations, such as the World Health Organization, the French Department of Health and Social Affairs, and the Urban Development Institute of Australia.

## Supplementary material

For supplementary material accompanying this paper visit https://doi.org/10.1192/bjb.2020.53. click here to view supplementary material
